# “Knowing It Before Blocking It,” the ABCD of the Peripheral Nerves: Part B (Nerve Injury Types, Mechanisms, and Pathogenesis)

**DOI:** 10.7759/cureus.43143

**Published:** 2023-08-08

**Authors:** Kartik Sonawane, Hrudini Dixit, Navya Thota, Tuhin Mistry, Jagannathan Balavenkatasubramanian

**Affiliations:** 1 Anesthesiology, Ganga Medical Centre and Hospitals, Coimbatore, IND; 2 Anesthesiology, Sir H. N. Reliance Foundation Hospital and Research Centre, Mumbai, IND

**Keywords:** reinnervation, nerve degeneration, nerve regeneration, pathophysiology of nerve injury, mechanism of nerve injuries, peripheral nerve injuries, nerve injuries

## Abstract

Selander emphatically said, “*Handle these nerves with care*,” and those words still echo, conveying a loud and clear message that, however rare, peripheral nerve injury (PNI) remains a perturbing possibility that cannot be ignored. The unprecedented nerve injuries associated with peripheral nerve blocks (PNBs) can be most tormenting for the unfortunate patient and a nightmare for the anesthetist. Possible justifications for the seemingly infrequent occurrences of PNB-related PNIs include a lack of documentation/reporting, improper aftercare, or associated legal implications. Although they make up only a small portion of medicolegal claims, they are sometimes difficult to defend. The most common allegations are attributed to insufficient informed consent; preventable damage to a nerve(s); delay in diagnosis, referral, or treatment; misdiagnosis, and inappropriate treatment and follow-up care. Also, sufficient prospective studies or randomized trials have not been conducted, as exploring such nerve injuries (PNB-related) in living patients or volunteers may be impractical or unethical.

Understanding the pathophysiology of various types of nerve injury is vital to dealing with them further. Processes like degeneration, regeneration, remyelination, and reinnervation can influence the findings of electrophysiological studies. Events occurring in such a process and their impact during the assessment determine the prognosis and the need for further interventions. This educational review describes various types of PNB-related nerve injuries and their associated pathophysiology.

## Introduction and background

Postoperative neurological symptoms due to nerve injuries can be present in up to 15% of patients [1]. It can become a nightmare for the patient and the involved clinical teams. Peripheral nerve injuries (PNIs) are a heterogeneous and distinct group of disorders resulting from various causes like vehicle accidents, falls, occupational injuries, domestic accidents, and penetrating trauma [2,3]. Apart from trauma, PNIs can also be caused by medical conditions (like diabetes, Guillain-Barre syndrome, or entrapment neuropathies), autoimmune conditions (like lupus, rheumatoid arthritis, or Sjogren’s syndrome), vascular pathologies, hormonal imbalances, or tumors. Although nerve injuries following peripheral nerve blocks (PNBs) are rare, they can be potentially devastating enough to arouse anxiety and affect an unfortunate patient’s overall quality of life (temporary or permanent). Reassuringly, most neuropathies appear transient rather than permanent, resolve over weeks or months, and rarely result in permanent injury.

The incidence of PNB-related transient neuropathies ranges from 0-2.2% at three months, 0-0.8% at six months, and 0-0.2% at one year [4]. Similarly, the incidence of long-term neuropathy after PNB is roughly estimated at <0.4% [5-7], while the range of permanent nerve injuries varies from 0.014% to 0.04% [4]. Such seemingly rare occurrences of PNB-related nerve injuries might be due to a lack of documentation (underreporting), improper follow-ups, or associated legal implications. Even the mildest, self-limiting, unintentional, and most frequent form of perioperative nerve injury (neuropraxia) can result in a medicolegal claim for extended hospitalization and additional treatment costs. Fear of such legal implications can lead to the underreporting of nerve injuries. However, timely diagnosis and treatment of any form of nerve injury are essential to avoid transient or permanent neurological damage and associated medicolegal claims. Proper documentation or reporting of unusual perioperative events indicative of possible nerve injury, detailed communication with the patient, and appropriate follow-up care can help control or minimize damages.

Nerve injuries can occur more frequently in the upper extremities than in the lower extremities. The most injured nerve is the radial nerve in the upper limb, followed by the median and ulnar nerves, and the sciatic nerve in the lower limbs, followed by the peroneal and tibial nerves [8]. It can be attributed to the number of blocks being performed more in the upper limbs than in the lower limbs and the increased protective connective tissue components in the nerves of the lower limbs compared to the upper limbs. Due to the varied neural-to-nonneural component ratio, the incidence of neurological symptoms after PNB varies with anatomical location, ranging from 0.03% for supraclavicular blocks to 0.3% for femoral blocks to 3% for interscalene blocks [9]. However, there is insufficient evidence to fully support this theory [4].

This article is part of a comprehensive overview of the essential understanding of peripheral nerves before blocking them. This educational overview will help readers understand the types, associated risk factors, mechanisms, and pathophysiology of nerve injuries that ultimately affect the overall outcomes and prognosis.

## Review

This narrative overview describes the various types, associated risk factors, mechanisms, and pathophysiology of nerve injuries. Related literature searches were performed using online platforms (PubMed, Medline, and Embase databases, Cochrane Library, and Google Scholar) using relevant search terms (neurons/nerve damage/nerve injuries/pathophysiology of nerve injury/nerve injury mechanisms/nerve injury risk factors/nerve degeneration/nerve regeneration/reinnervation). Articles published in English were selected, and their reference sections were manually searched for additional information.

Legalities of iatrogenic nerve injuries

Iatrogenic (also known as iatrogenous or iatropathic) nerve injuries are always a cause for concern as they are caused by the treating physician, related personnel, or circumstances during the treatment process. When a patient comes into the hospital with no nerve damage and comes out with nerve damage, a charge of negligence can seldom be resisted [10]. Although they make up only a small portion of medicolegal claims, they are difficult to defend. Even with the NHS Litigation Authority, most PNIs cases are recorded as surgical or procedural complications but not under the heading of PNIs [11]. Trauma surgeries pose a specific risk for iatrogenic nerve injuries due to associated distorted anatomy, fracture malalignment, hematoma formation, bleeding, and disrupted soft tissue planes. The iatrogenic PNIs can be classified into Type 1 to Type 3 depending on the associated causes (Table 1) [12,13]. PNIs can occur in any surgical procedure under GA and RA. Despite perioperative causes, preexisting conditions like diabetes, smoking, hypertension, and neuropathy also contribute to PNIs and further complicate diagnosis, management, and prognosis.

**Table 1:** Classification of iatrogenic peripheral nerve injuries.

Classification of iatrogenic peripheral nerve injuries	
Type 1	When the injured nerve is not involved in surgery/procedure or when operating in proximity to the nerve, or with inexperienced/poor technique.
Type 2	When the injured nerve is involved in surgery/procedure. During nerve decompression procedures like carpal tunnel release.
Type 3	When the injured nerve is the target for the repair of different nerves during nerve harvest for grafting

The most common allegations are attributed to insufficient informed consent; preventable damage to a nerve or nerves; delay in diagnosis, referral, or treatment; and misdiagnosis and inappropriate follow-up treatment [10]. The essential requirements, therefore, must include acquiring a thorough knowledge of nerve anatomy and the pathophysiology of nerve injuries. Also, all PNIs should be promptly identified, accurately diagnosed, properly documented, and, in substance, discussed or communicated with the patient. With any anesthetic or surgical procedure, more focus or attention should be paid to prerequisites, keeping charts, and following preventable measures to avoid nerve injury. Without such measures, defending against a claim against PNIs is very difficult. As PNIs are essentially avoidable, following all preventable measures for preventing nerve injuries at each stage should be part of standard anesthetic care.

Anatomy and histopathology of peripheral nerves

**Figure 1:**
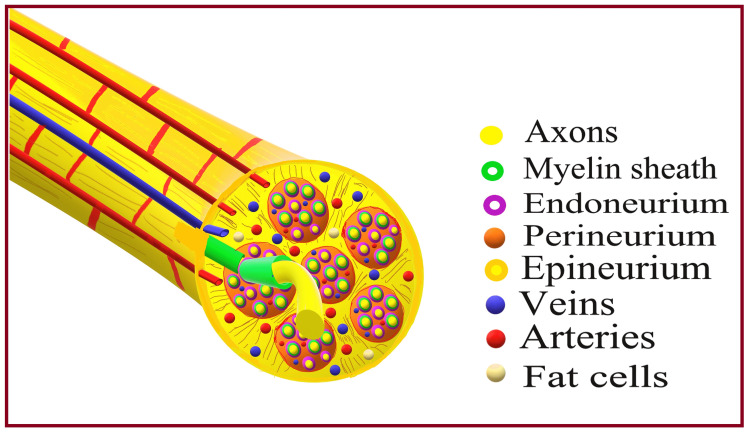
Peripheral nerve and its connective tissues. Source: This figure was created by the first author (KS).

Any pathology affects the normal functional anatomy and physiological processes of the structure. Therefore, it is essential to understand the normal functional anatomy and physiology of the nerve before knowing the pathogenesis of nerve injuries. Nerve anatomy and physiology have already been described in Part A of this review article. As mentioned in that part, each nerve has neural and non-neural components (Figure 1). Neural components comprise numerous nerve fibers arranged in different bundles called nerve fascicles. A delicate tissue matrix known as the endoneurium surrounds each nerve fiber in the fascicle. A tough epithelial covering called the perineurium held all the fascicles together. A loose collection of collagen fibers known as the epineurium supports and encases all fascicles. The degree of functional and anatomical disruption of neural and non-neural components of the nerve determines the nature and prognosis of nerve injury. However, certain risk factors play an important role in causing such neural insults.

Risk factors contributing to nerve injury

**Figure 2:**
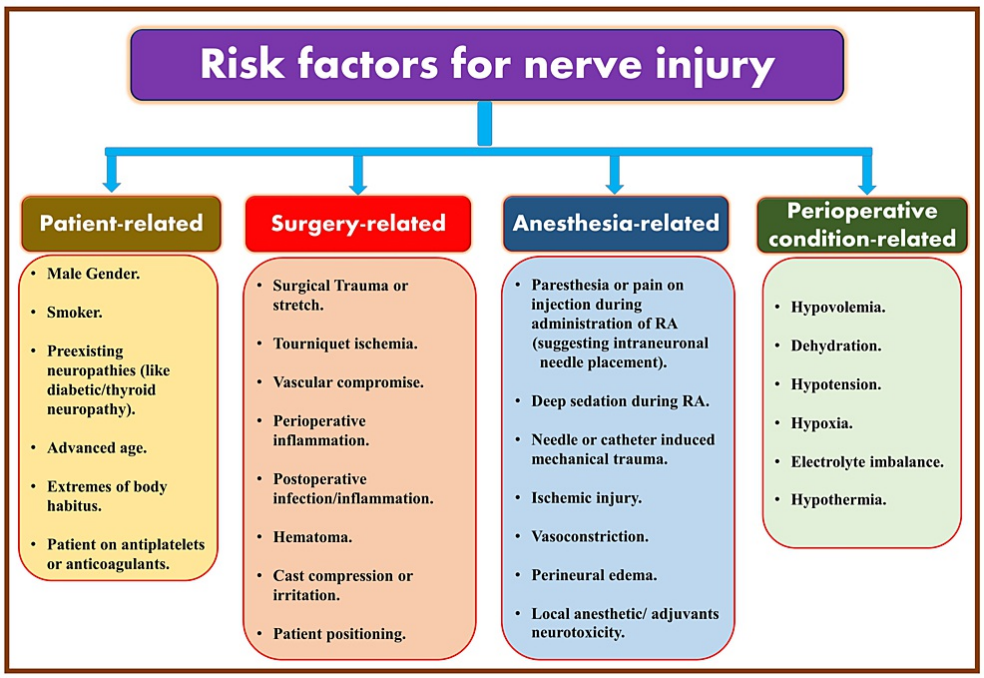
Associated risk factors of nerve injuries. DM: diabetes mellitus; RA: regional anesthesia. Source: This figure was created by the first author (KS).

The potential etiologies causing nerve injury or contributing factors are categorized into patient-related, surgical-related, anesthesia-related, and perioperative conditions-related risk factors (Figure 2) [14-16]. Nerve injuries in the perioperative period can have a variety of causative factors determined by the timing of their occurrence. Postoperatively, nerve injuries can occur within 48 hours of surgery and last up to three weeks. Early nerve injury within 24 hours of surgery can be due to wound hematoma, edema, or nerve laceration. The delayed injuries (days to weeks later) can be due to scar formation or tissue reactions.

**Figure 3:**
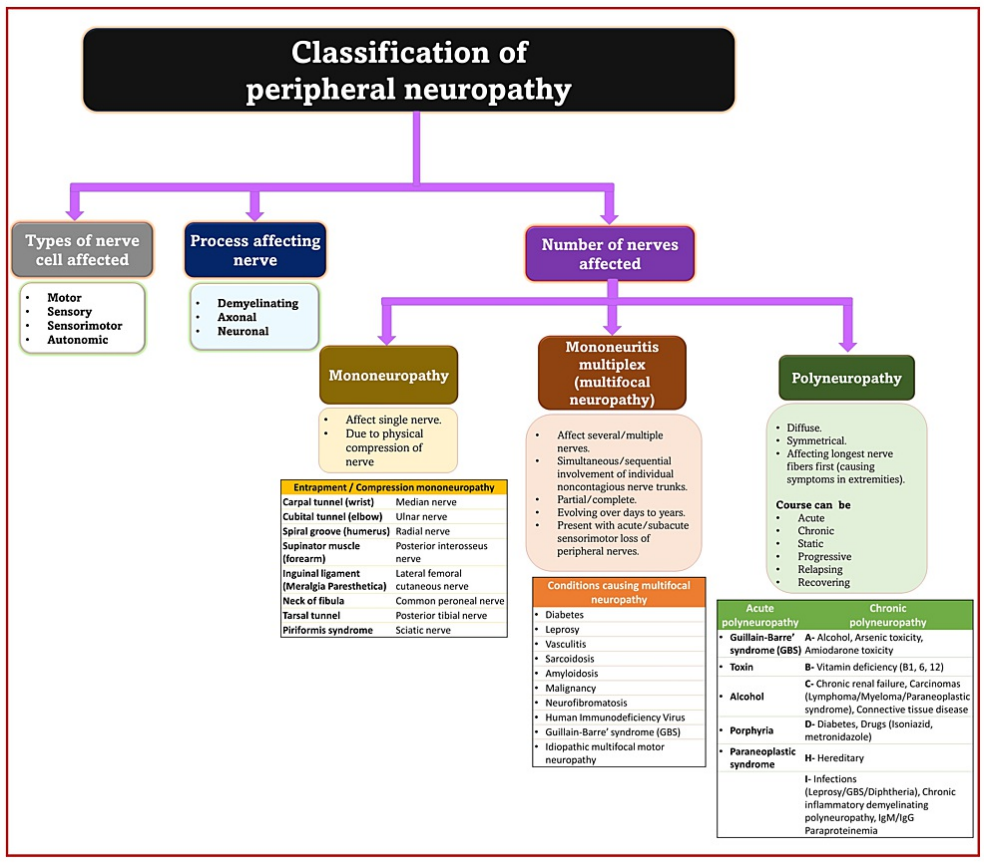
Classification of peripheral neuropathies. Source: This figure was created by the first author (KS) [14-16].

Various preexisting conditions and comorbid factors can cause damage to single nerves (mononeuropathy) or many nerves (polyneuropathy). Depending on the number of nerves involved, the type of neurons affected, and the processes involved, peripheral neuropathies are classified in many ways (Figure 3) [17-19]. Metabolic neuropathy encompasses a broad spectrum of peripheral nerve disorders associated with systemic disorders of metabolic origin. These diseases include, but are not limited to, diabetes mellitus, hypoglycemia, uremia, hypothyroidism, liver failure, polycythemia, amyloidosis, acromegaly, porphyria, lipid/glycolipid metabolism disorders, nutritional/vitamin deficiencies, and mitochondrial disorders. The common hallmark of these diseases is the involvement of peripheral nerves by altering the structure or function of myelin and axons due to metabolic pathway dysregulation. Among the various metabolic diseases mentioned, diabetic and thyroid disorders affecting peripheral nerves individually or in combination are common. Controlling diabetes or correcting thyroid hormones helps correct neuropathy promptly, reducing the risk of neurological injury [20].

Diabetic neuropathy

**Figure 4:**
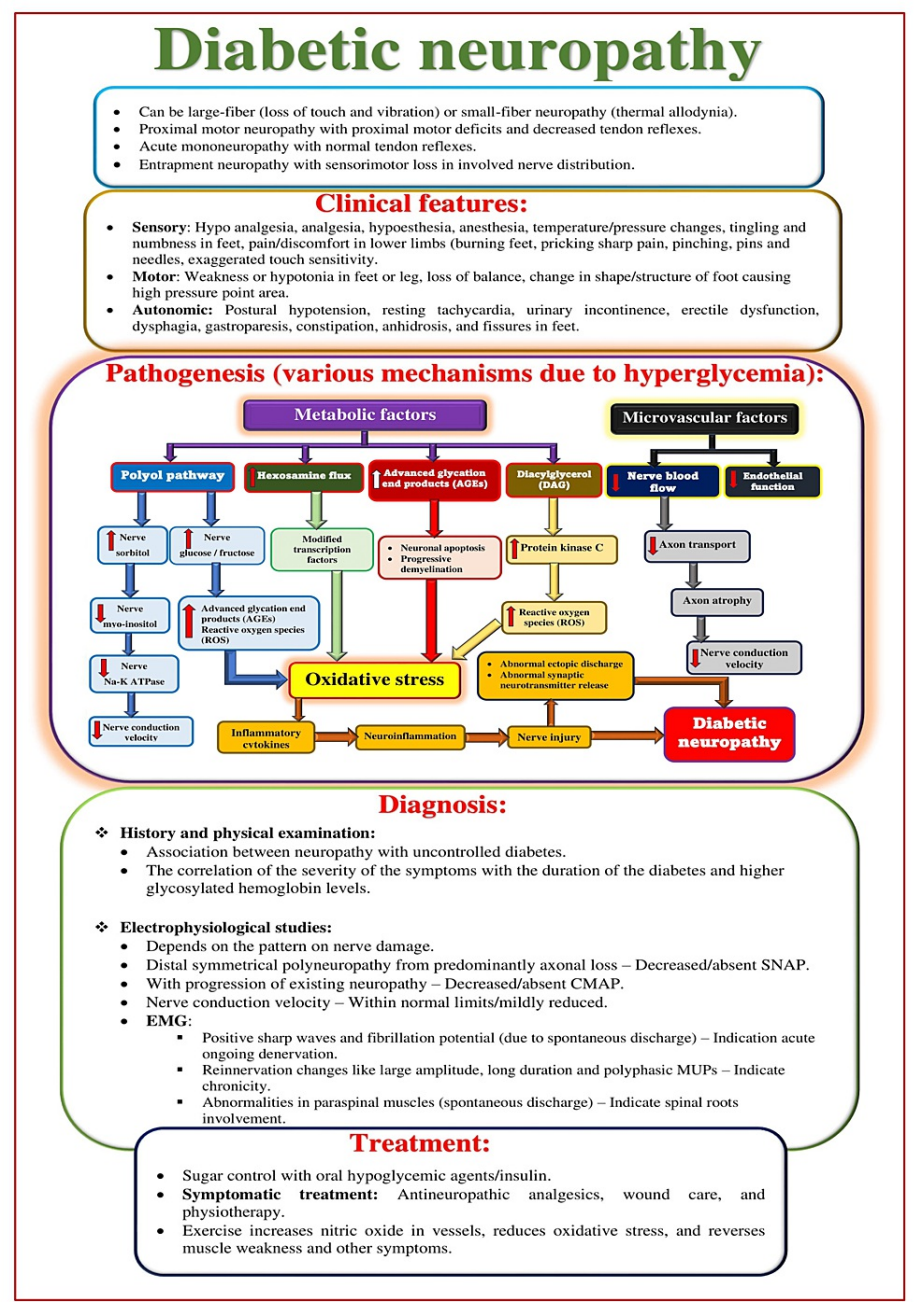
Overview of diabetic neuropathy. SNAP: sensory nerve action potential; CMAP: compound muscle action potential; EMG: electromyography; MUPs: motor unit potentials. Source: This figure was created by the first author (KS).

Diabetic peripheral neuropathy (DPN) is the most common complication of diabetes (Figure 4) [21,22]. It involves loss of myelin sheath integrity, Schwann cell apoptosis, and endoneurial microangiopathy [23]. It can be attributed to prolonged hyperglycemia [24], dyslipidemia, rising serum uric acid levels [25], smoking [26], and vitamin D deficiency [27]. Hyperglycemia in diabetes mellitus (DM) can result in increased flux of polyol pathways (in Schwann cells), decreased production of neurotrophic factors (myelin, ciliary neurotrophic factor (CNTF), nerve growth factor (NGF), and neurotrophin-3 (NT-3)), mitochondrial dysfunction, oxidative stress, altered lipid metabolism, the release of inflammatory factors leading to neuroinflammation, axonal demyelination, and a slow nerve conduction velocity (NCV) [28,29]. The severity of the DPN can be correlated with the duration of diabetes and glycated hemoglobin (HbA1c) [30-33]. Higher HbA1c levels (>6.5%) indicate a 16.9-fold likelihood of developing DPN [34].

Schwann cells are responsible for neuronal growth and regeneration by maintaining neuronal structure and function, nourishing axons, and promoting survival and repair after injury [28]. Schwann cell damage may represent the first step in DPN pathogenesis, leading to segmental axonal demyelination and myelin regeneration, followed by axonal degeneration [28]. It affects myelinated and unmyelinated nerve fibers and involves sensory, motor, and autonomic nerves. The impaired vasculature and autoregulation due to long-term hyperglycemia can lead to hypoxic nerve damage involving distal-predominant nerve fiber degeneration. The most distal axons of small fibers distribute in the skin epidermis and can sense pain and prick. DPN significantly involves these nerve fibers, resulting in distortion, twisting, focal swelling or beading, and finally, the disappearance of nerve fibers [35-37].

Thyroid neuropathy

**Figure 5:**
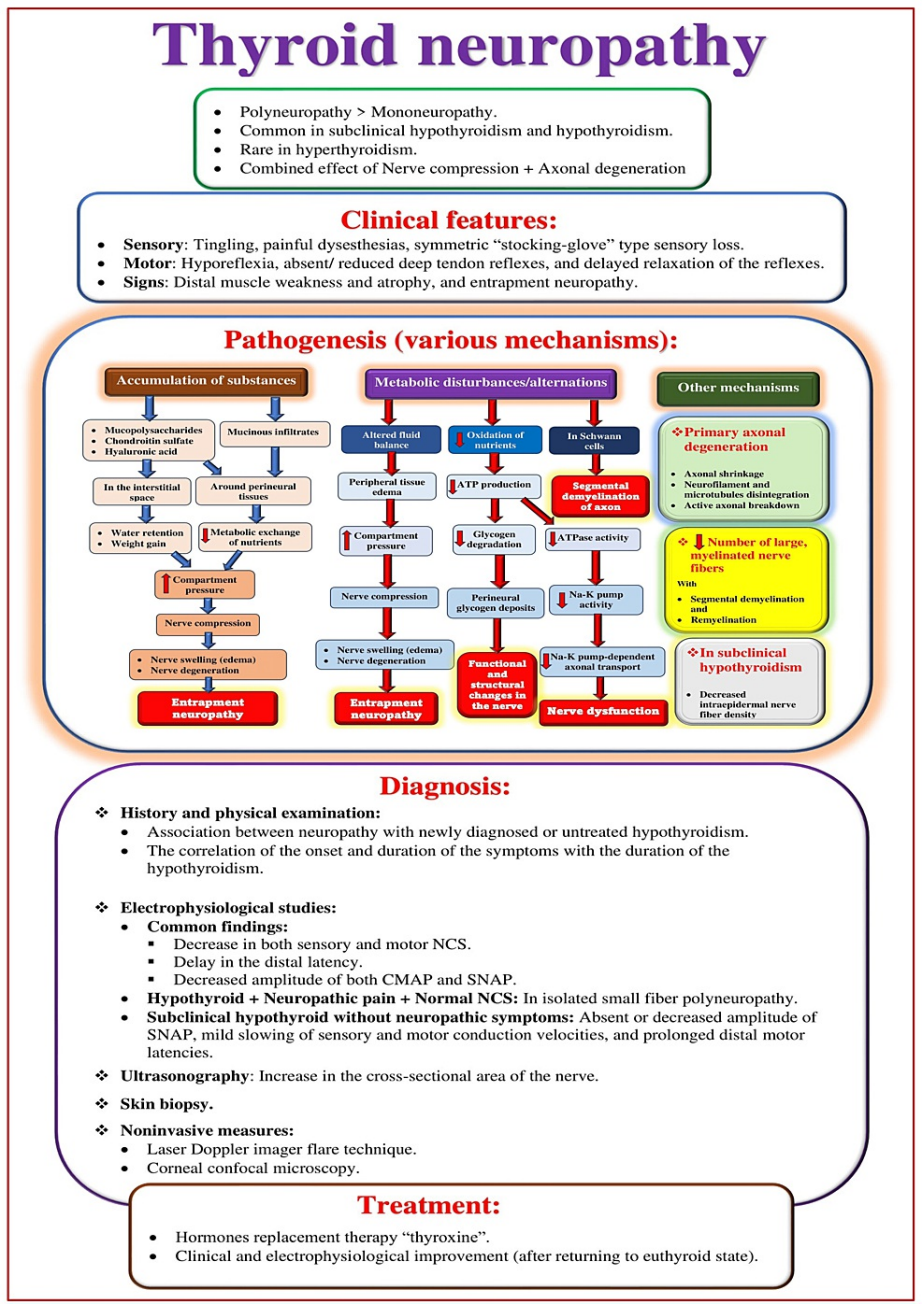
Overview of thyroid neuropathy. NCS: nerve conduction studies; CMAP: compound muscle action potential; SNAP: sensory nerve action potential. Source: This figure was created by the first author (KS).

Thyroid hormones significantly affect normal tissue growth, metabolism, and development. They regulate many functions and processes of the nervous system and exert various effects on the neuromuscular system and the brain [38]. Therefore, neuromuscular dysfunction can be observed in 20%-80% of thyroid diseases [39]. Hypothyroidism can involve both central and peripheral nerves [40]. Neuropathy symptoms include loss of reflexes, proximal muscle weakness, numbness, paresthesia, decreased sensations, and slowed muscle contraction and relaxation [38]. Developing thyroid neuropathy (Figure 5) can be a combined effect of nerve compression/entrapment and axonal degeneration. Carpal tunnel syndrome is common, with hypothyroidism resulting in damage to the median nerve [41]. Sensory polyneuropathy is associated with distal sensory loss and reduced/absent deep tendon reflexes. Polyneuropathy with defects in the nerve cell body, axon, or myelin sheath shows decreased nerve conduction velocity and amplitude.

Thyroid dysfunction and DM

**Figure 6:**
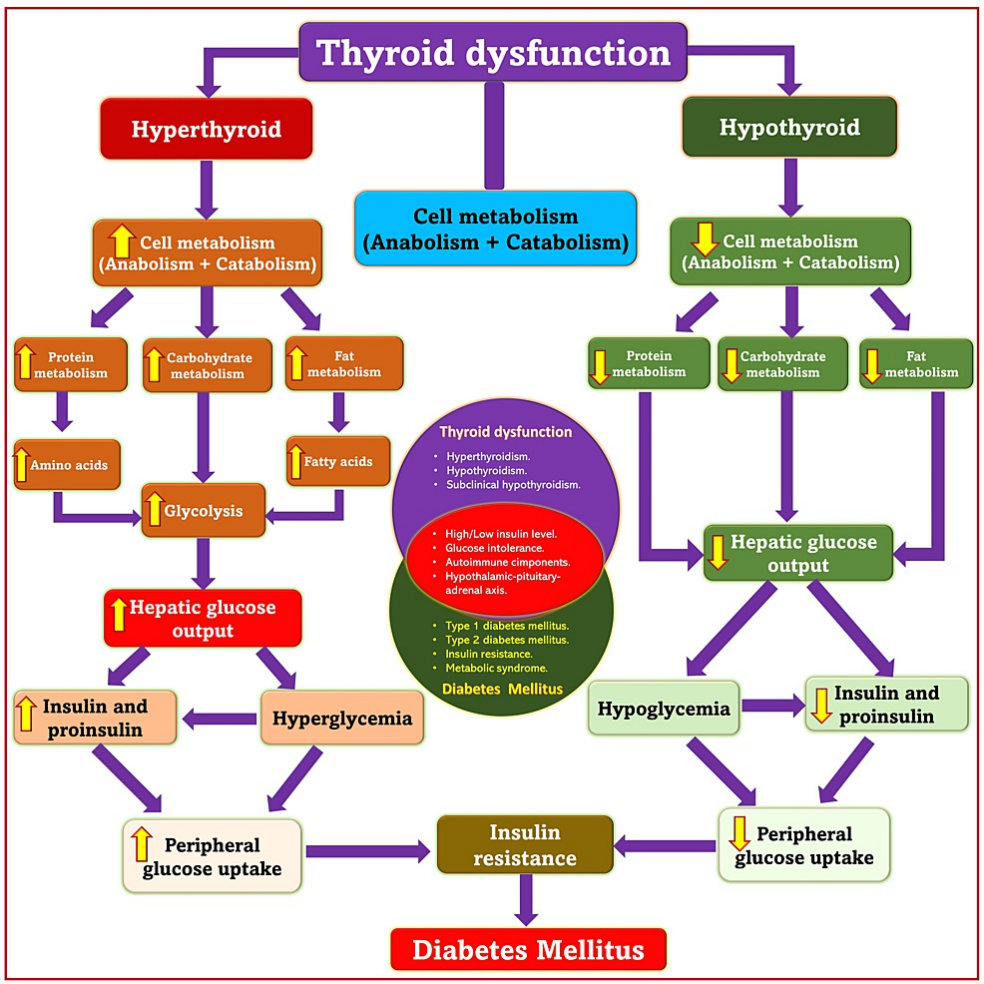
Interlinking between thyroid dysfunction and diabetes mellitus. Source: This figure was created by the first author (KS).

DM and thyroid dysfunction are common endocrinopathies in adults that always interact and frequently coexist (Figure 6). The pathophysiological association between the two is reflected in a complex set of interlinked biochemical, genetic, and hormonal factors [42-43]. A possible mechanism could be the complex interaction between the signaling pathways associated with glycometabolism and insulin resistance [44-46] and hyperlipidemia [47] with higher serum thyroid-stimulating hormone (TSH) levels. Subclinical hypothyroidism is the most frequently encountered thyroid disease [48,49] and is characterized by normal levels of thyroid hormone (T3 and T4) and elevated levels of TSH. Elevated serum TSH levels are also linked with incidences of type 2 DM [50,51]. Both clinical and subclinical hypothyroidism can produce insulin resistance, leading to the development of type 2 DM. TSH can exacerbate the abnormal metabolism of glucose and lipids. It can also significantly exacerbate DPN, possibly through Schwann cell oxidative stress and apoptosis due to TSH receptors palmitoylation [28].

Studies have shown a very high prevalence of thyroid dysfunction in type 2 DM. Therefore, routine determination of thyroid hormone levels is recommended in diabetics whose conditions are difficult to treat. Both hyperthyroidism and hypothyroidism carry the risk of glucose intolerance [52]. Nearly 50% of Graves’ disease patients have varying degrees of glucose tolerance, and 2-3% develop overt diabetes. Hyperthyroid patients with DM may have worsening glycemic control, and thyrotoxicosis may trigger diabetic ketoacidosis due to elevated lipolytic actions and increased hepatic β oxidation [53,54]. Insulin and thyroid hormones are closely involved in cell metabolism. Therefore, an excess or deficiency of either can lead to dysfunction of the other [52]. Hyperthyroidism promotes hyperglycemia [55], and hypothyroidism can be associated with recurrent hypoglycemic episodes [56]. Thyroid hormone replacement has been shown to reduce fluctuations in blood glucose levels [57].

Double crush syndrome

The presence of an underlying chronic neuropathy resulting from mechanical (compression), ischemic (peripheral vascular disease), toxic (cisplatin chemotherapy), or metabolic (diabetes) disease may predispose patients to further neurological injury [58]. Such a condition is known as the double crush syndrome/phenomenon [58,59]. The double crush phenomenon can also be observed in nerve entrapment syndromes, such as carpal tunnel syndrome (median nerve) or cubital tunnel syndrome (ulnar nerve). It suggests that patients with preexisting neural impairment may be more susceptible to injury elsewhere when exposed to secondary insults like various concomitant risk factors related to patient, surgery, or anesthesia. Two minor injuries along a peripheral nerve trunk are worse than a single site. However, the damage from the double injury far exceeds the expected additional damage caused by each isolated injury [59]. The second injury need not be along the periphery of the nerve trunk but at any point along the nerve transmission pathway. Therefore, performing neuraxial as well as peripheral regional anesthesia (RA) techniques in patients with preexisting neurological disorders may theoretically put them at increased risk of the double crush phenomenon. In comparison, the triple crush phenomenon can be observed in conditions like diabetics, where high compartment pressure of the leg causes nerve and soft tissue injuries. It represents existing neuropathy (diabetic neuropathy) with an ongoing process causing it and an increased susceptibility to further insult [60].

Triple crush syndrome

Triple crush/triple hit injury or syndrome is rarely described in a few anecdotal reports. Like double crush syndrome described for conditions like carpal tunnel/ cubital tunnel syndrome, triple crush syndrome incidences have been reported in a few cases where nerve insults occur at three different sites. These cases include diabetic polyneuropathy (causing tibial nerve damage) [60], cervical disc degenerative disease with persistent phrenic nerve palsy (during interscalene block) [61], radial nerve injury due to insults at three sites (humerus level, supinator syndrome, cheiralgia paresthetica) [62], and three-site compressions in patients with lumbar spinal stenosis with foraminal, extraforaminal, and lateral recess compression [63]. As previously mentioned, a double crush is the compression and pinching of a nerve in two places, or an existing damaged nerve that suffers another hit/crush/injury, leaving the proximal or distal portion of the nerve vulnerable to further injury. Similarly, the triple crush phenomenon denotes a triple hit, injury, or nerve compression at two sites where an additional impairment, such as compression/needle trauma/pressure due to injection volume, surgery, or anatomy, is added to resultant neurological damage. Thus, the three sites or modes of injuries act synergistically to produce nerve damage and associated symptoms.

Proposed mechanisms of pathophysiology of triple crush syndrome include impairment of axonal transport, up- and down-regulation of ion channels, and inflammatory changes in the dorsal root ganglion [64]. The volume of local anesthetic has been postulated as one of the possibilities of persistent diaphragmatic paresis due to phrenic nerve involvement. It has also been hypothesized that in severe diabetic neuropathy, there is an increase in compartment pressure which can further lead to nerve and soft tissue damage. Therefore, a triple crush can be a source of nerve injuries that need to be identified or anticipated. Careful documentation and assessment of patients with preexisting and severe neuropathy (e.g., degenerative diseases of the lumbar spine or diabetic/thyroid polyneuropathy) particularly contribute to the prevention, risk reduction, treatment, prognostication, and medicolegal aspects pertaining to peripheral nerve injury due to PNBs. Using USG with the minimum required volumes of LAs and vasoconstrictors can help mitigate nerve injury in patients having a risk of double crush or triple crush. Before administering PNB, screening for preexisting neuropathic conditions is necessary to avoid further catastrophes and to treat such patients by applying all preventive measures to avoid nerve injury.

Relevance to the practice of RA

Diabetic patients are at least twice as likely to require surgery than non-diabetics because of their comorbidities and the type of surgery performed [65]. They are predestined for many interventions under RA [66]. Although the RA technique offers numerous advantages over GA, its use in patients with preexisting neuropathy is controversial. The concern raised includes an increased risk of nerve injury and difficulty accurately detecting an intraneural needle placement (owing to decreased sensations and paresthesia). The altered nerve physiology in diabetic neuropathy can lead to altered nerve excitability [67,68]. The stimulation threshold is related to the membrane properties of the peripheral nerve [68,69]. The 0.3-0.5 mA stimulating threshold current is considered sufficient to elicit a motor response without discomfort or minimal discomfort to the patient [70]. However, threshold or even higher currents cannot preclude the placement of an intraneural needle [71-76].

The higher stimulating current requirement in diabetics can be due to decreased nerve conduction velocity, action potential amplitude, and excitability. Involved mechanisms include mitochondrial oxidative dysfunction, oxidative stress, inflammation, decreased Na-K-ATPase activity, loss of myelinated fibers, and demyelination [66]. These are attributed to the insufficient function of peripheral blood vessels, changes in immunity and metabolism, and changes in sodium-calcium channel expression [67,77,78]. Keyl et al. found a geometrically higher mean threshold current in diabetic patients (1.9 mA current) than in non-diabetic patients (0.26 mA current) during popliteal nerve block [79]. Similarly, Bigeleisen et al. demonstrated a higher median stimulation threshold in diabetic patients (1.3 mA) than in non-diabetic patients (0.5 mA) during supraclavicular brachial plexus block [72]. Some diabetics with severe neuropathy may require an unusually higher current of >3mA to elicit a motor response despite the needle being close to the nerve [66]. The higher current of 2.4 mA sometimes failed to elicit a motor response despite clear ultrasound detection of needle-nerve contact [72,79].

Therefore, the choice of stimulating current can be particularly important when dealing with a patient with preexisting neurological deficits (like diabetic or thyroid neuropathy). The intensity and the duration of the current required for nerve stimulation are increased. Hence a setting in the PNS (amplitude and pulse width) has to be modified accordingly. An experienced anesthetist should do a nerve block when performed. Landmark-based or paresthesia-based techniques are not recommended. Such neuropathic conditions can pose a high risk of intraneural needle placement and subsequent nerve damage when PNS is used alone. Therefore, techniques like dual guidance (USG +PNS) or triple guidance (USG + PNS + injection pressure manometer) can be better options to prevent nerve injuries and provide safe RA. Details of these techniques are included in the next part (part C) of this review article [80].

Types of nerve injuries

**Figure 7:**
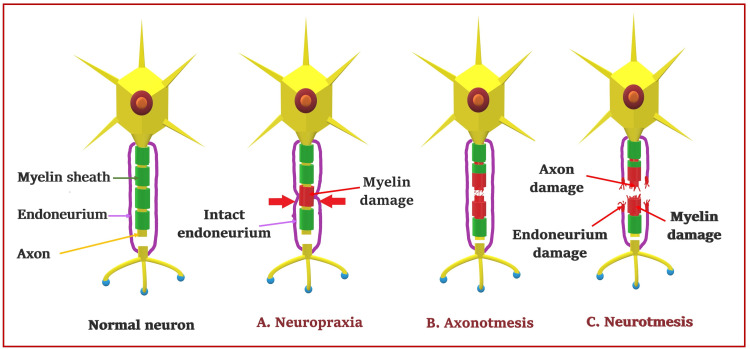
Types of nerve injuries. Source: This figure was created by the first author (KS).

**Figure 8:**
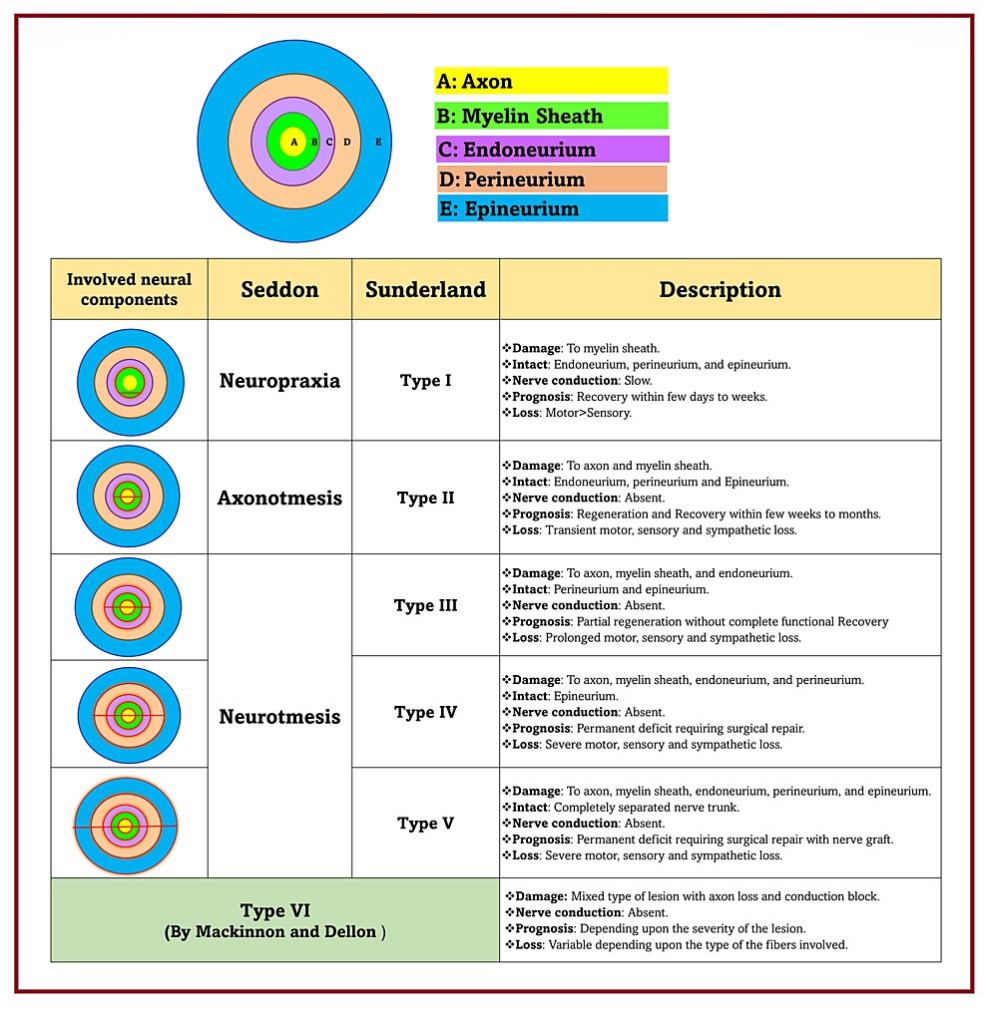
Classification of nerve injuries and schematic representation of involved components. Red color (line/borders): damaged components of the nerve. Source: This figure was created by the first author (KS).

Nerve injury classification depends on the affected neural components, functional disruption, and neural recovery potential. In 1942, Seddon (a Professor of Orthopaedics) classified peripheral nerve injuries in English as a transient block, lesion in continuity, and division of nerve. Seddon classification is based on the degree of functional disruption [81,82]. His colleague Henry Cohen, a neurologist, renamed it in Greek as neuropraxia, axonotmesis, and neurotmesis (Figure 7). In 1951, Sunderland (a professor of anatomy) disliked using Greek, assuming modern doctors would not understand it [83]. Therefore, he proposed a numbered classification (Grade I-V) based on the increasing involvement of the neural connective tissue (Figure 8). Type I (Grade I) degree of nerve injury of Sunderland corresponds to the neuropraxia of Seddon’s classification. However, the comparison of other degrees of Sunderland with axonotmesis and neurotmesis in the literature is ambiguous and variable. Sunderland gradings are based on the concept that neural connective tissue elements are important for regeneration. Its increasing involvement in successive degrees of nerve injury can also impact the prognosis from good to bad to worst. With increasing severity of nerve injuries (III -V), the disrupted connective tissues result in the “cross-wiring” of the regenerating axons. It further leads to the misinterpretation of nerve signals and the misfiring of the wrong muscles.

Similarly, in 1988, Lundborg (a surgeon) [84] classified seemingly innocuous nerve injuries (like neurapraxia) into type A and type B based on physiological conduction block. Type A injury is associated with transient intraneural circulatory arrest resulting in the metabolic block without nerve fiber pathology. It can be immediately reversible. Type B injuries are associated with more severe nerve damage, such as intraneural edema, resulting in increased endoneurial fluid pressure. It may be reversible within days or weeks after correcting the cause. In 1989, Mackinnon and Dellon introduced another sixth-degree (Type VI) in the Sunderland classification (Figure 8) as a mixed lesion with axon loss and conduction block [85]. This type of injury is probably considered the most common nerve injury. Birch and Bonney (1991) also classified nerve injuries into degenerative and non-degenerative lesions [86]. The Seddon classification is easier to follow among all the classification systems above. While surgeons more commonly use the Sunderland grading to decide on nerve repair [87].

Neuropraxia (Transient Block)

Neurapraxia is the mildest type of nerve injury associated with reversible conduction blockade due to localized myelin sheath degeneration or segmented demyelination. Such injury can be seen due to nerve compression leading to transient ischemic damage to the myelin sheath without affecting the axon and neural tube/connective tissues. Neuropraxic injury secondary to transient or intermittent ischemia improves quicker than injury secondary to focal demyelination. It predominately affects large nerve fibers more than small fibers [3]. Focal demyelination leads to leakage of current at the nodes of Ranvier, resulting in impaired impulse conduction across the nerve segment with transient weakness or paresthesia. Severe demyelination can lead to a complete conduction block causing physiological damage in the anatomically intact nerve.

Perioperative neurapraxia occurs mainly due to improper patient positioning or poor padding of the pressure points leading to entrapment neuropathies (carpal/cubital tunnel syndrome). Such compression injuries can involve the ulnar nerve (at the elbow), radial nerve (at humerus), sciatic nerve (lithotomy position/hyperflexion of hip), or peroneal nerve (against the fibular head). LA can also cause neuropraxic injury by causing vasoconstriction, direct neurotoxic effect, or mechanical pressure effect due to LA administration. The inflammatory reactions during PNB can also cause scar formation and subsequent nerve entrapment. Other mechanisms include crush, traction, ischemia, thermal, electric shock, radiation, percussion, and vibration [88,89]. Details of various neuropraxic injury mechanisms are discussed later in this article.

In neurapraxia, compound muscle action potential (CMAP) and sensory nerve action potential (SNAP) can be elicited distal to the injury. However, due to partial or complete conduction block proximal to the injury, varying degrees of decreased CMAP and SNAP amplitudes and decreased conduction velocity can be recorded in nerve conduction studies (NCS). Such changes improve completely or partially upon completion of remyelination. Electromyography (EMG) does not reveal any abnormal spontaneous activities in the form of fibrillations or positive sharp waves. It also shows normal morphology and an absent or reduced number of motor unit potentials (MUPs) on voluntary contractions. The prognosis is good, with usually complete recovery within days to weeks (12 weeks) [90]. Details of NCS and EMG studies are discussed in Part D of this review article.

Axonotmesis (Lesion in Continuity)

Axonotmesis is a more severe type of nerve injury than neuropraxia. The complete interruption of axons in axonotmesis can cause conduction loss in the nerve fiber beyond the zone of injury. It involves axonal damage without affecting neural connective tissues (endoneurium, perineurium, and epineurium). Such injury is common with crush and stretch injuries [91]. It can also occur in nerves involved in displaced bone fractures. Injury to the axons results in separation from the cell body, disrupting the transport of essential molecules and the electrical signal from the cell body to the axon fragment [92]. Loss of electrical responsiveness occurs due to swelling of an axon fragment (due to a rise in the calcium levels) [93] and subsequent fragmentation of both the axon and myelin. Neuronal death is possible if the injury is more proximal and close to the cell body. In such injury, reinnervation, recovery, and motor unit potential (MUP) will be seen.

Recovery in axonotmesis involves collateral sprouting and regeneration. Collateral sprouting occurs if less than 20-30% of the motor axons are involved [94]. It takes about 2-6 months [94]. The degree of recovery depends on the injury site and the amount of fibrosis that occurs. Regeneration occurs if more than 90% of the axons are involved [95]. It requires an intact myelin/Schwann cell tube to support the growth of the axon. These tubes are viable for 18-24 months following injury [96]. Axon regeneration needs to occur within this period; otherwise, the myelin tubes degenerate and interrupt the regeneration process [96].

NCS shows conduction loss due to demyelination in the distal segment (3-4 days after injury) and small or absent CMAP or SNAP due to axonal loss. EMG studies show spontaneous electrical activities in the form of fibrillation potentials or sharp waves (2-3 weeks after injury) due to axonal loss leading to muscle denervation. The denervated muscles can subsequently become atrophic. A good prognosis with almost complete recovery can be possible without surgery if the causative factors that lead to nerve damage are removed in time. Recovery takes weeks to months (up to 18 months), involving axonal regrowth at a rate of 1 mm/day or 1 inch/month [97].

Neurotmesis (Division of Nerve)

Neurotmesis is the most severe form of nerve injury associated with the complete transection of the nerve. It is often observed after massive trauma, sharp injuries, traction or avulsion injuries, and the injection of noxious drugs [98]. It involves damage to the neuronal (axons and myelin sheath) and nonneuronal (nerve connective tissues) components. NCS indicates demyelination (distal conduction loss) and axonal loss (absent CMAP/SNAP). EMG studies show spontaneous electrical activities indicating axonal loss and muscle denervation. Recruitment of MUPs on voluntary contraction is usually absent, but its presence indicates axonal sparing. The degree of involvement of connective tissues determines the recovery potential of neurotmesis. The growth is fair if only endoneurium is involved and poor when both endoneurium and perineurium are involved. However, there is no growth if all neural connective tissues (endoneurium, perineurium, and epineurium) are involved with neuronal elements. Therefore, the recovery can be unpredictable. However, surgical repair involving direct approximation of the two segments or nerve grafting [99] is essential to enhance reinnervation and recovery. The subsequent recovery can take anywhere from two to 18 months, depending on the severity and site of the lesion.

Mechanisms of the nerve injuries

**Figure 9:**
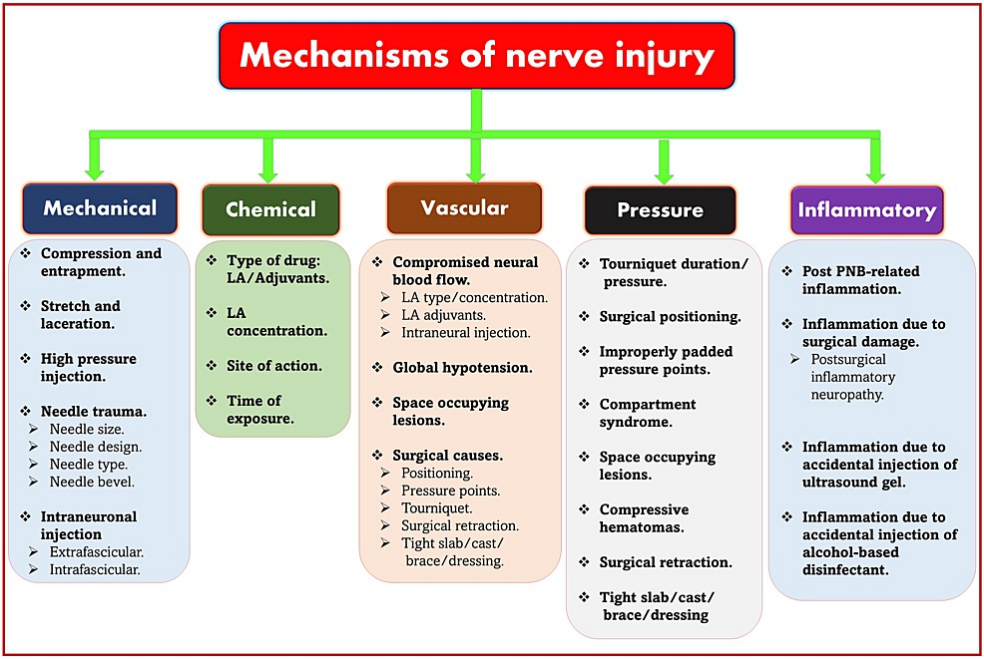
Various mechanisms of nerve injury. LA: local anesthetic. Source: This figure was created by the first author (KS).

Nerve injuries are multifactorial in origin. Various mechanisms (Figure 9) can be responsible for PNB-related nerve injuries that can occur simultaneously or in combination. However, the exact etiology of perioperative nerve injuries remains unclear in many instances. Suggested etiologies include mechanical trauma from the needle, nerve edema and/or hematoma, pressure effects of the local anesthetic (LA) solution, neurotoxicity of the injectate (LA and adjuvants) [100], and nonspecific inflammation following surgery or PNB. Other important and avoidable causes of nerve injury are compression secondary to hydraulic pressure (tourniquets) within a rigid compartment or by overextension or flexion of limbs for prolonged periods. Confounding factors that may play a role in nerve injury include pre-existing neuropathies (like diabetes or thyroid neuropathies), surgical manipulation, prolonged tourniquet pressure, or compression from postoperative bandages/casting/slab/brace [101].

Mechanical Trauma

Mechanical trauma can cause neuropraxic injury to the nerve through compression and entrapment, leading to ischemia. On the other hand, other severe forms of nerve injury due to stretch and lacerations can also be possible. High injection pressure can increase intrafascicular pressure, which can lead to ischemic nerve damage if exceeded. Mechanical nerve injury can also be possible due to direct needle trauma or intraneuronal injections. Penetration of any needle or subsequent injection into the nerve can inevitably lead to inflammation and cellular infiltration, regardless of whether the clinical injury occurs.

Needle Trauma

**Figure 10:**
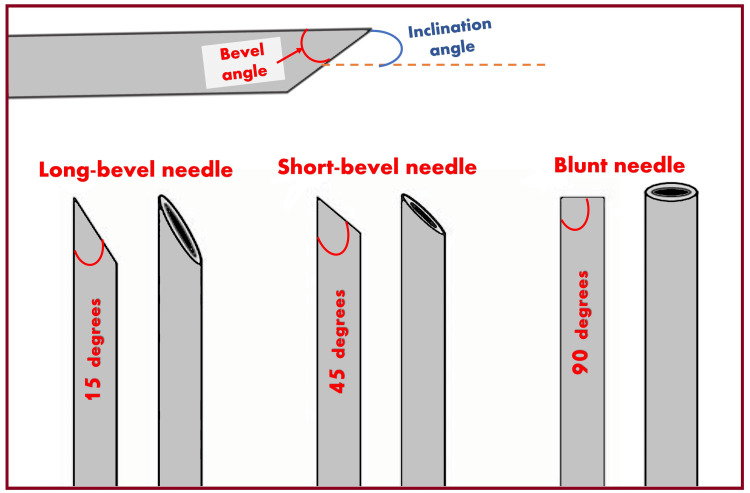
Types of needle tips as per bevel angle. Source: This figure was created by the first author (KS).

The characteristic of the mechanical injury caused by the needle depends on the needle size and type, especially the angle at the needle’s tip (bevel angle). The effect of needle size also affects the severity of the injury - smaller needles (24 gauge) may lead to less nerve injury than larger needles (19 gauge) [102]. Needle tip characteristics (Figure 10) also influence the likelihood of fascicular penetration [103]. It is believed that short-beveled (blunt) needles cause less damage (Table 2) than long-beveled (sharp) needles. However, such assumptions remain unsupported by the available literature. Various cellular changes are associated with needle trauma: Alterations in membrane channel expression, activation of signal transduction, neuropeptide production, and overall increased excitability of the dorsal horn [104].

**Table 2:** Comparison between long-bevel needles and short-bevel needles.

Sharp tip/long bevel (12^o^-15^o^) needle	Blunt tip/short bevel (>45^o^) needle
Nerve penetration chances are more and deeper.	Nerve penetration chances are low and less deep.
Produce cleaner and more likely-to-heal cuts.	Produce noncongruent cuts and more extensive damage.
Recovery is faster and more complete.	Recovery is slower and incomplete.
More likely to enter fascicles but causes less extensive damage.	Less likely to enter the fascicle but, if entered, causes more extensive damage.
The sharp tip can bend and form a microscopic hook when it hits a hard surface like a bone, causing more nerve damage when the needle is pulled out and moved.	The blunt tip cannot form a microscopic hook upon hitting the hard surface.
The partial insertion of a sharp-tip needle into the nerve is less likely to cause nerve injury due to the more elongated oval exit portion, which remains outside the nerve.	With the blunt-tipped needle, the entire lumen is more likely to be embedded in the nerve structure, resulting in the drug being injected at the intraneural level.

Intraneuronal Injection

**Figure 11:**
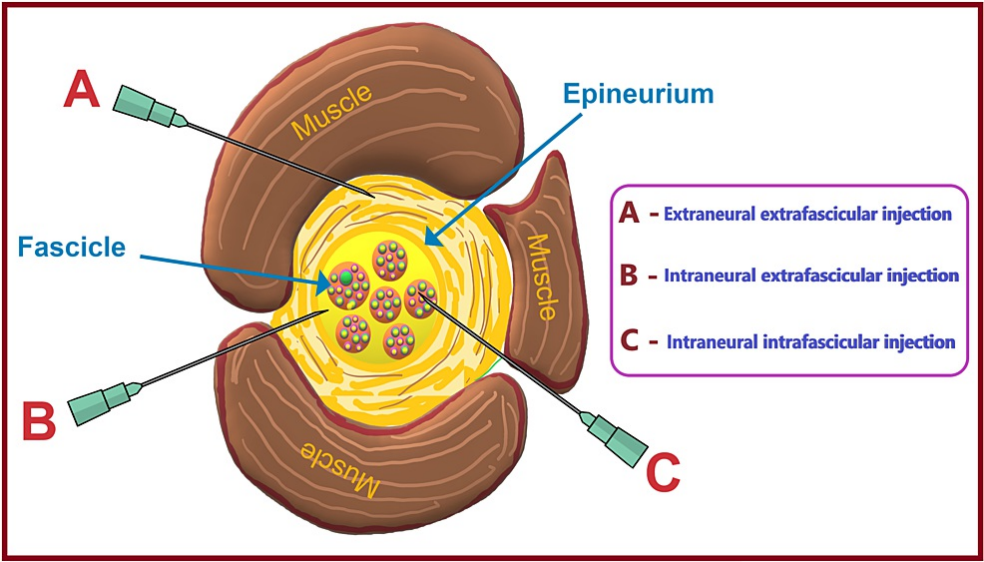
Locations of extraneural and intraneural injections. Source: This figure was created by the first author (KS).

Intraneural needle placement (Figure 11) can be extrafascicular (within the loose epineural connective tissue but outside the perineurium) or intrafascicular (within the tough perineural sheath covering the fascicle). Compact fascicular assembly is less likely to be penetrated by an advancing needle. However, once penetrated, it can result in a wide and multifactorial spectrum of injury due to the needle itself or the injectate. As peripheral nerves move away from the neuraxis, the ratio of nonneuronal-to-neural components within the nerve tends to increase. The risk of an intrafascicular injection differs from site to site in the peripheral nervous system and correlates with the cross-sectional fascicle-epineurium ratio.

The perineurial multilayered epithelial lining does not readily stretch to accommodate increased intrafascicular pressure. Such increased intrafascicular pressure upon injection may remain higher than capillary perfusion pressure leading to neural ischemia and inflammation [105]. The mechanical disruption of the perineurial sheath may result in injury to the axons and/or the leakage/herniation of endoneurial contents [106]. Therefore, an intrafascicular injection of even very small amounts of LA can lead to widespread axonal degeneration and permanent neural damage. In contrast, extrafascicular injections into the compliant epineural space appear to be associated with a minimal increase in pressure. It is due to the lax and accommodating stromal architecture of epineurium, leading to a lower likelihood of disruption of normal nerve architecture [107,108].

Chemical Mechanism

**Figure 12:**
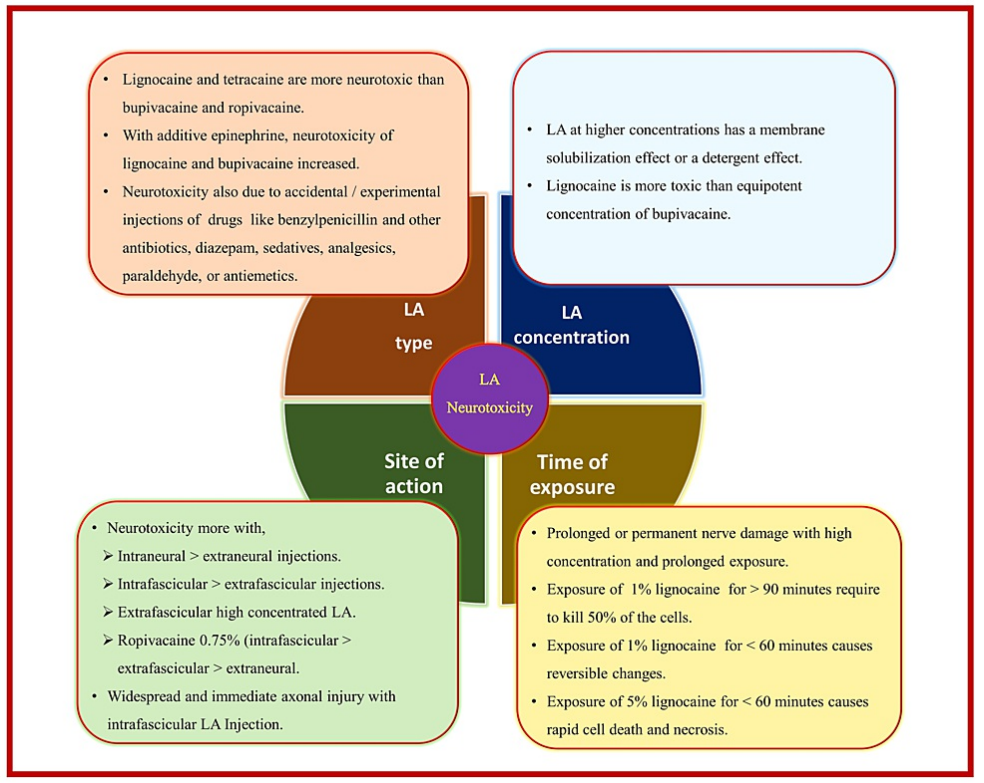
Local anesthetic (LA) neurotoxicity. LA: local anesthetic. Source: This figure was created by the first author (KS).

It includes the toxicity of injected solution containing either LA alone or a combination of LA and adjuvant. All LA agents are known to have a neurotoxic effect on the nerve that can occur even at clinical concentration levels [107,109]. Neurotoxicity increases further when the injection site is particularly intraneural or intrafascicular. Proposed mechanisms of neurotoxicity include increased intracellular calcium concentration, disturbance in mitochondrial function, interference with membrane phospholipids, cell apoptosis, and inhibition of cell growth, motility, survival, and morphological changes [100,110-112]. Vasoconstrictive properties of LA as well as adjuvants such as adrenaline, increase the risk of ischemic nerve damage [100]. Oxidative damage after nerve ischemia and reperfusion leads to the initiation of apoptosis by affecting Schwann cells. The extent of neural damage by LA depends upon the type, concentration, site of action, and exposure time (Figure 12) . Newer LA perineural drug delivery systems such as liposomal, microspheres, and cyclodextrin are being introduced to prolong the analgesic effect [113-115]. However, there are also concerns about their potential neurotoxic effects due to compounds, metabolites, and liposomal core degradation [116].

Various LA adjuvants have been used in RA with mixed success. These include alpha-2 adrenoceptor agonists (clonidine and dexmedetomidine), steroids (dexamethasone), anti-inflammatory drugs (parecoxib and lornoxicam), midazolam, ketamine, magnesium sulfate, and neostigmine. The concerns about the safety profile of these adjuvants due to their potential for neurotoxicity and neurological complications warrant further investigation [117]. Adrenaline potentiates the effects of LA due to its alpha-2-mediated antinociceptive property and prolongs the duration of blockade by decreasing systemic absorption of LA due to its vasoconstrictor property. However, potential neurotoxicity can be observed with the perineural use of adrenaline, particularly in patients with diabetes mellitus, hypertension, and smokers [118,119]. Dexamethasone has been found to increase levels of the enzyme serine-threonine protein kinase B (aka Akt) and lead to attenuated neurotoxicity of bupivacaine and lidocaine [120]. Akt protects against apoptosis under various conditions, such as glutamate toxicity or oxygen or glucose deprivation. Pharmacological inhibition of Akt can abolish this protective effect of dexamethasone [121]. The use of neuraxial or perineural nonsteroidal anti-inflammatory drugs as adjuvants has been of great concern due to their histopathological evidence of neurotoxicity [122,123]. The neurotoxicity of perineural neostigmine is dose-dependent, with doses below 50 μg considered safe [124,125]. Ketamine [126] and midazolam [127] are not recommended for use in PNB due to proven neurotoxic effects.

LA neurotoxicity can be concentration-dependent. Therefore, low-concentration LA can be considered safe in clinical practice. The neuraxial use of 5% lignocaine has been discontinued due to the occurrence of cauda equina syndrome. The recommended concentration of bupivacaine is 0.5%, whereas bupivacaine 0.75% has been abandoned in Europe [122]. Evidence suggests that lignocaine is more toxic than equipotent concentrations of bupivacaine [128,129]. Ropivacaine is considered a safe local anesthetic [130,131]. Its S-enantiomer is associated with lower lipid solubility and offers a superior neurological and cardiac toxicity profile compared to an equal dose of bupivacaine [132]. However, in vitro and in vivo studies suggest the neurotoxic potential of ropivacaine [133,134] can be due to apoptosis via regulation of the Akt pathway.

Vascular Mechanism

The vascular mechanism includes factors that can cause ischemic nerve damage by impairing neuronal blood flow. Such factors include the needle or injectate, which may contain LA or adjuvants. The vascular systems in the peripheral nerve include extrinsic or epineurial blood vessels and intrinsic or endoneurial blood vessels connected by oblique trans-perineurial blood vessels. Damage to these blood vessels by needle can result in external or internal hematoma formation. The compressive effect of the hematoma and subsequent edema formation can lead to neural ischemia due to reduced neural blood flow.

LA alone can also decrease the dose- and concentration-dependent neural blood flow. Various concentrations of levobupivacaine and ropivacaine can significantly reduce sciatic nerve blood flow in rats [135,136]. Adrenaline is a common adjuvant used to prolong block duration and as a vascular marker [137]. Adding adrenaline can enhance ischemia due to vasoconstriction and reduced blood flow (Table 3).

**Table 3:** Effect of various concentrations of adrenaline (epinephrine) on neural blood flow.

Concentration of adrenaline	Effect on neural blood flow
2.5 μg/ml	20% Transient Increase.
5 μg/ml	20% Reduction.
10 μg/ml	35% Reduction.
2% lignocaine+ 5 μg/ml adrenaline	80% Reduction.

Intraneural but extrafascicular injection of LA can cause altered perineurial permeability and fascicular edema. It can lead to fascicular compression and decreased neural perfusion. Whereas intraneuronal and intrafascicular injection can cause a prolonged increase in endoneurial pressure. It can result in endoneurial ischemia if the increased endoneurial pressure exceeds capillary perfusion pressure. The earliest response to nerve ischemia is depolarization and the generation of spontaneous activity that is symptomatically perceived as paresthesia. It can also cause loss of all sensations with the onset of limb ischemia. If the ischemic time is less than 2 hours, nerve function can return within 6 hours. If the ischemia lasts up to 3 hours, the nerve undergoes edema formation and a degeneration phase of 1-2 weeks, followed by a regeneration phase of up to 6 weeks [138].

Pressure Mechanism

The pressure mechanism indirectly involves reduced blood supply to the nerve due to increased pressure in the structure around the nerve. Such increased pressure can result from improperly padded pressure points during patient positioning, compressive lesions such as hematoma or edema around neural structures, and using tourniquets. The femoral, ulnar, peroneal, lateral cutaneous nerve of the thigh and the brachial plexus are at risk of compression due to over-extension or flexion and inadequate padding during prolonged surgery. Compressive hematomas are most common in patients treated with anticoagulants [139,140]. In contrast to a spinal or epidural hematoma, peripheral neuropathy from compressive hematoma typically resolves completely [141-144].

**Figure 13:**
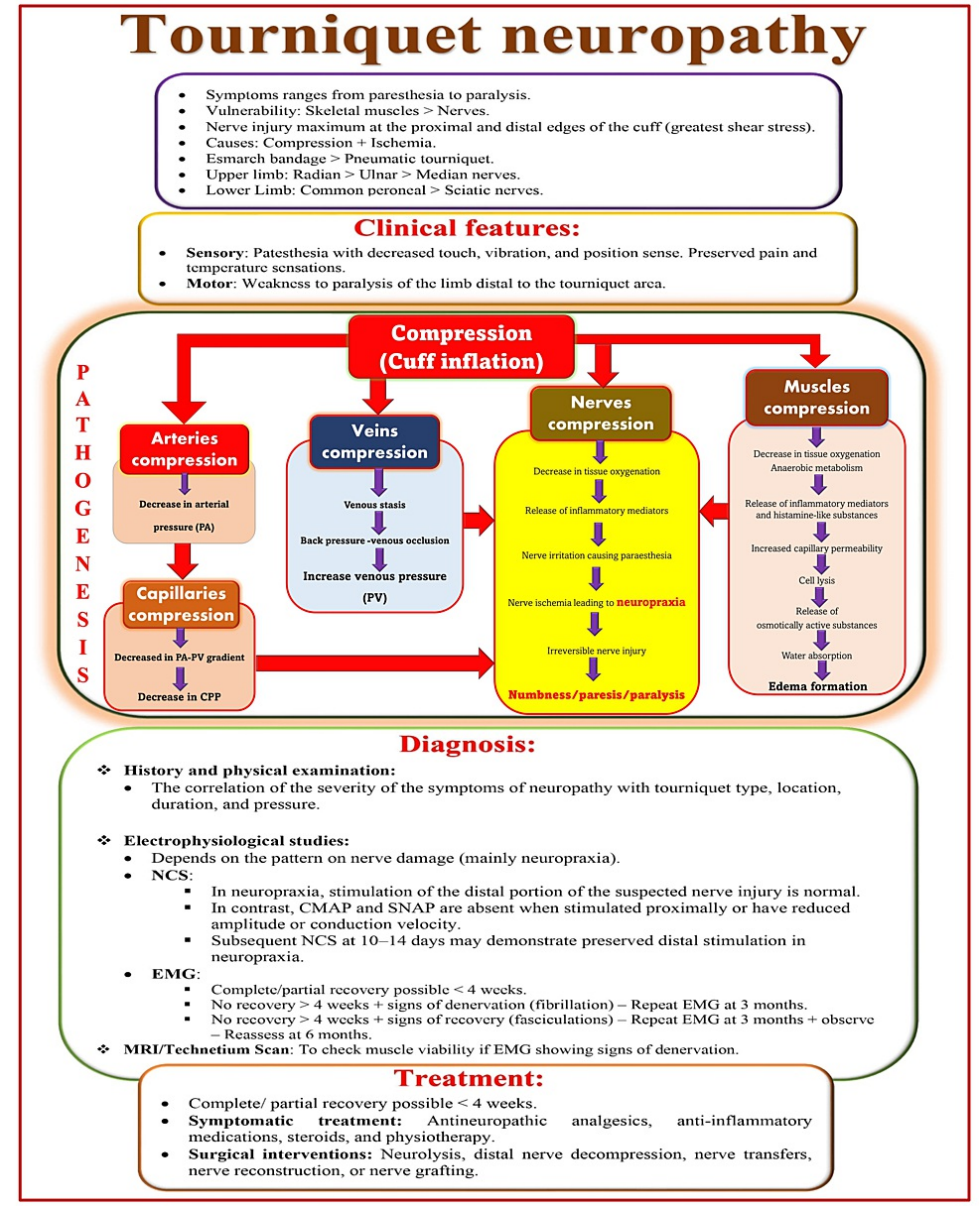
Overview of tourniquet neuropathy. CPP: capillary perfusion pressure; NCS: nerve conduction studies; CMAP: compound muscle action potential; SNAP: sensory nerve action potential; EMG: electromyography. Source: This figure was created by the first author (KS).

Tourniquet neuropathy (Figure 13) can occur with an incidence of 1 in 8000 cases [145]. It can range in severity from neuropraxia to permanent neurological damage. The tourniquet can cause nerve damage either by mechanical deformation or ischemia [145-150]. Tourniquet neuropathy typically involves weakness or paralysis and decreased sense of touch, vibration, and position. However, pain and temperature (heat and cold) sensations remain preserved [151]. Its occurrence can be directly correlated to the duration of the tourniquet and the pressure applied. According to the recommendations, the tourniquet pressure should not be more than 150 mmHg above the systolic blood pressure, and the duration should not exceed 90-120 minutes without a 10-15 minute deflation phase [152]. The limb occlusion pressure (LOP) is determined using Doppler to find the optimal cuff pressure. LOP is the minimum pressure required to stop arterial blood flow in the limb distal to the cuff. The optimal cuff inflation pressure can be calculated by adding 50 mm Hg in LOP for the upper limb and 75 mm Hg for the lower limb [153]. Esmarch bandages are not recommended as they can generate very high pressure. Wider cuffs than narrow cuffs are preferred as they generate lower pressure. Despite adhering to all recommendations, nerve injuries such as neuropraxia can develop in patients with preexisting neuropathy [154,155].

Inflammatory Mechanism

Inflammation after PNB or surgery can also affect a nerve at the same or distant site, causing neurological deficits. Studies have reported signs of chronic inflammation, such as adhesions, fascia thickening, vascular changes, and scar tissue formation that cause compression neuropathy in PNB [156]. Such inflammation can also be caused by accidental injection of ultrasound gel [157-159] or alcohol-based disinfectant (used for surface disinfection) around the nerve during PNB. Neuropathy caused due to inflammation following surgery is called postsurgical inflammatory neuropathy (PSIN). It has typically delayed onset, may be focal or multifocal, remote from the surgical site, and is associated with pain and weakness in the area of the affected nerves. In PSIN, the existing pathology and tissue damage associated with the surgery stimulates the immune response, manifesting as neural tissue inflammation [160,161]. Axonal degeneration and lymphocyte-mediated inflammation indicate such an inflammatory-immune mechanism. Affected nerves show signs of edema, microvascular disruption, myelin injury and loss, and axonal injury with the influx of acute inflammatory cells [160,161]. Such neuropathy can occur at the surgical site or remotely on the same limb or other body regions. It can also occur in multiple locations.

PSIN can be definitively diagnosed by a nerve biopsy. However, magnetic resonance imaging (MRI) can support diagnosis and, together with the clinical evidence, enable a preliminary diagnosis and therapy [160]. Magnetic resonance neurography (MRN) can provide additional information about the severity and extent as well as the location of the nerve lesion [162]. Electrophysiological studies can assess the severity of a nerve injury. Such a condition can gradually improve over time. In some cases, corticosteroids can help, but the condition remains permanent sometimes [163].

Pathogenesis after nerve injury

A complex multi-cellular response occurs when an axon in the peripheral nervous system (PNS) is injured. Any injury to the nerve can lead to the interruption of axonal continuity (axotomy), dividing it into two cytoplasmic compartments: proximal and distal. The proximal portion of the axon that remains in continuity with the cell body (soma) is the proximal nerve stump. The detached portion of the axon from the soma is the distal segment. The cell body begins to express regeneration-associated genes. The proximal segment forms a growth cone moving toward the denervated target, and the distal segment degenerates. Such degeneration and regeneration occur in injured nerves and nonneuronal cells, including Schwann and immune cells.

**Figure 14:**
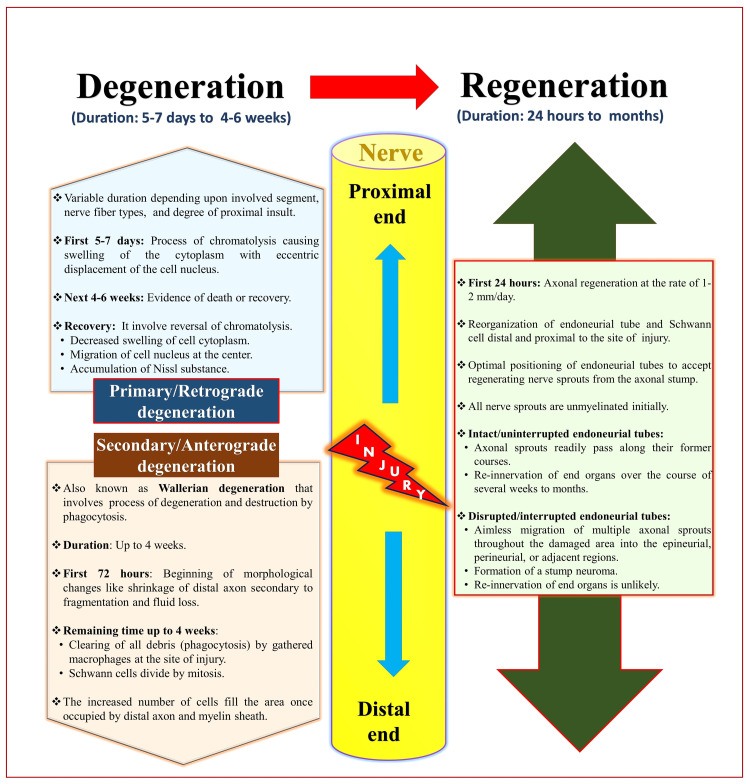
Degeneration and regeneration following nerve injury. Source: This figure was created by the first author (KS).

Any nerve injury follows the process of degeneration followed by regeneration (Figure 14) [164]. Degeneration can occur proximal as well as distally to the site of injury. Degeneration at the proximal site of injury is known as primary or retrograde degeneration, whereas at the distal site of the injury, it is known as secondary, anterograde, or Wallaerian degeneration [164].

Primary Degeneration

It is also called retrograde degeneration. It proceeds for at least one internodal space and heavily depends on the degree of proximal insult. The cut end of the proximal segment gets retracted from the injury site, known as proximal segment dieback [165]. Morphological changes in the parent cell body vary with the cell type and its proximity to the injury site. The cell body responds to nerve injury by chromatolysis [165], which includes the formation of cytoplasmic vacuoles, nucleolar enlargement, eccentric displacement of the nucleus, and cell body swelling.

Secondary Degeneration

It is also known as anterograde or Wallerian degeneration, which occurs in the distal segment. It involves degeneration and destruction by phagocytosis and lasts about four weeks [165]. This biphasic process involves an initial latent interval of 36-44 hours post-injury, during which the distal axonal segment remains intact and excitable [166]. It follows an abrupt and complete axonal fragmentation phase, reaching totality as quickly as one hour from the onset. Secondary degeneration involves multiple processes like axonal discontinuity, disruption, degradation, dissolution, demyelination, and denervation.

The axonal discontinuity followed by axonal transport disruption due to nerve injury results in an unregulated influx of calcium ions [165]. Subsequently, the calcium-dependent proteolytic enzymes promote the degradation of the axonal cytoskeleton and its membrane proteins [165]. It also leads to the dissolution of microtubules and neurofilaments. Ultimately, the breakdown of axolemma integrity contributes to axon fragmentation and collapse. The structural disruption of the nerve ending coincides with the loss of physiological activity. The concomitant loss of presynaptic terminals leads to transmission failure and the cessation of spontaneous end-plate activity. However, end-plate function can persist for 8-10 hours after nerve injury [165].

**Figure 15:**
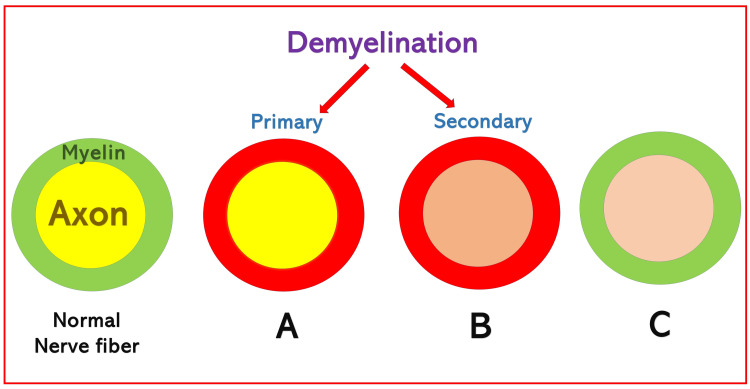
Types of demyelination of the nerve. (A) Primary demyelination with involvement of myelin without axon. (B) Secondary demyelination with involvement of both myelin and axons. (C) Involvement of the axon without myelin (seen in inflammatory changes in disease or trauma). Source: This figure was created by the first author (KS).

The degeneration of the axon leads to demyelination (Figure 15) due to the involvement of the Schwann cells. Such simultaneous degeneration of the surrounding myelin sheath with axonal degeneration is called secondary demyelination. In contrast, primary demyelination (in diseases like multiple sclerosis) involves isolated myelin breakdown around the intact axon. Isolated axonal injury without impact on myelin can be seen in inflammatory changes during the disease process or trauma.

Neuronal Death

Some axotomized neurons survive but become atrophied due to the limited anabolic response, leading to neuronal dysfunction. In contrast, some axotomized neurons undergo regressive changes leading to neuronal death. Some neurons that cannot survive axonal damage succumb to what is referred to as reverse cell death [167]. Cell death following PNI depends on several variables, like the types of affected neurons, their proximity to the cell body, the type of injury, and age-related factors. Following an injury, the surviving neurons shift their metabolism towards the biosynthesis of cell survival and repair molecules. It results in the upregulation of the production of certain cytoskeletal and growth-related proteins. Cell death can occur in neurons with lesions near the cell body of origin (“anterograde (orthograde) transneuronal” or “trans-synaptic degeneration) or in neurons that form a synapse with the damaged neurons (“retrograde transneuronal”/“retrograde transsynaptic degeneration) [165].

Regeneration

The neuronal regeneration process is impossible in the central nervous system [168]. It can be due to slower clean-up, inhibition by oligodendrocytes, or an unsuitable environment. At the same time, regeneration in the PNS is crucial and is enabled due to faster clean-up by macrophages and facilitation by Schwann cells. It cannot be possible when the cell body is damaged, but it is possible with axonal damage. It depends on the intrinsic properties of the affected neurons and the extrinsic molecular and cellular influences of the surrounding nonneural structures. Many factors initiate and influence the regeneration process: Upregulation of growth-promoting genes (responsible for axonal regrowth) following nerve injury, inflammatory response to nerve injury, and wound healing by macrophages via the production of growth factors and certain cytokines (with neuronal growth-promoting properties) [165,169]. Regeneration involves the healing and growth of damaged axons, followed by remyelination and reinnervation.

The proximal nerve segment forms multiple sprouts from each damaged axon with an amoeboid structure at the end known as a growth cone [170]. The formation of such growth cones represents the earliest phase of axonal regeneration. These growth cones are initially exposed to the cellular and molecular microenvironment of the lesion site. Proliferating cells from the endoneurium and epineurium of the proximal and distal stumps and other surrounding nonneural tissues infiltrate the wound site and form a tortuous terrain. The likelihood of regenerating fibers reaching the distal nerve stump decreases as the distance between lesions increases.

Schwann cells also begin to proliferate in the distal nerve stump within the first 24 hours after injury and reach a peak of mitotic activity after about three days [165]. After 3-5 days, the regenerating fibers reach the distal stump, meeting the terrain of linearly oriented Schwann cells. The plasma membrane of the Schwann cell has growth-promoting and cell-adhesive molecules that play important roles in nerve regeneration and remyelination. Regenerated axons are usually much smaller in diameter and have a thinner myelin sheath than the parent axon. Functional recovery is highly dependent on the ability of the axons to reconnect to their targets, i.e., reinnervation. Any disruption of the continuity of this internal architecture through injury can cause misdirection of regenerating axons while entering the distal stump, leading to reinnervation failure.

The total time for regeneration depends on the healing time and dieback of the proximal nerve segment, the navigation of growth cones through the lesion to the distal stump, and the distance traveled by regenerating axons to reach their target. Nerve regeneration proceeds at 3-4 mm/day after crush [171] and approximately 2-3 mm/day after transection injury. Whereas axonal regrowth proceeds at 1-2 mm/day or 1 inch/month [165,172]. The delay in regrowth depends on the severity of the nerve lesion and its proximity to the cell body. The likelihood of reinnervation of viable muscle decreases when the injury is far from the target. It can result in muscle atrophy due to loss of neurotrophic support and functional disuse. Therefore, regeneration rate and successful reinnervation are often major constraints on optimal functional yields.

## Conclusions

In summary, in-depth knowledge of the types and pathophysiology of nerve injuries can help to understand the extent and significance of adopting preventive measures in the perioperative period. In addition, various mechanisms of nerve injury provide clues that help anticipate their possibilities and encourage vigilant monitoring at every step in the perioperative period. PNB-related PNIs can be linked with the needle type/design, LA type/volume/concentration, adjuvant, target site, or administration techniques. For ensuring safe RA, the goal should be to select the safest modality and appropriate LA solutions (as per requirements), understand the existing damages of the preexisting conditions, and remain extraneural or extrafascicular. Time-lapse of pathologic processes after injury (like degeneration, regeneration, remyelination, or reinnervation) can help prognosticate nerve injuries and ascertain appropriate treatment protocols. However, current research on PNIs presents a challenge, as exploring such PNB-related nerve injuries in living patients or volunteers may be unfeasible or unethical. Additionally, the surgical outcomes of nerve repairs are still in the gray zone, warranting further research into newer modalities to achieve the desired functional outcomes.
